# Parricide as a typical offence guided by psychosis – a retrospective analysis.

**DOI:** 10.1192/j.eurpsy.2026.11276

**Published:** 2026-07-31

**Authors:** V. Watzal, S. Klug, A. Dvorak

**Affiliations:** 1 Department of Psychiatry and Psychotherapy; 2 Comprehensive Center for Clinical Neurosciences and Mental Health, Medical University of Vienna, Vienna; 3 Forensic Therapeutic Center Göllersdorf, Göllersdorf, Austria

## Abstract

**Introduction:**

Parricide is a rare homicide offence with a worldwide proportion of 2 to 4% (Heide *et al*. J InterpersViolence 2007; **22**, 1382–1399; Heide *et al*. Oxford University Press 2013). In individuals with psychotic disorders this percentage increases to 20%. This highlights a certain clinical and criminological implication for those mentally ill offenders currently scantily investigated.

**Objectives:**

Accordingly, our aim was to evaluate preceding psychopathological, psychosocial and offence-related factors in a forensic parricide-offender group in a Central European specialized institution for mentally ill perpetrators.

**Methods:**

All adult parricide-offenders, who were not guilty by reason of insanity and/or treated as a forensic patient between January 1998 and March 2023 at the Forensic Therapeutic Center Göllersdorf were included in the present study. Data on psychopathological, psychosocial and criminal offence parameters were extracted from their medical and forensic history as well as court documents. Data was analyzed with descriptive statistics.

**Results:**

In total, 26 adult parricide-offenders were included in the present study (14 completed parricides, 10 attempted parricides, 2 both). 92.3% of all parricide offenders were not guilty by reason of insanity (§21/1 according to Austrian criminal code) and 7.7% were culpable but were under the influence of a severe mental disorder (§21/2 according to Austrian criminal code). 84.6% of the patients were diagnosed with a schizophrenia disorder while only one out of 26 patients suffered from bipolar disorder. The mean age at the time of the offence was 30.5±8 years. 50% of the cases were a patricide while 38.5% of the offences can be defined as a matricide. In 5 out of 26 cases the offence had multiple victims. In 53.9% of the cases a knife was used as a weapon, blunt force trauma, gunshot wounds and strangling were each present in 7.7% of the offenders. Delusional misidentification was detected in 32% of the patients’ psychopathological examinations, command auditory hallucinations in 24%, poisoning ideas in 20% and feelings of being threatened by their parents in 32% of all cases.

**Conclusions:**

A cluster of parricide cases committed by young adults with psychotic disorders consists of an escalation of a symptom complex containing delusional misidentifications, command auditory hallucinations, poisoning ideas and threat perceptions against their immediate family members. Further research should focus on additional risk factors, preventive treatment and measures.

**Disclosure of Interest:**

None Declared

